# Inverse association between serum insulin and sex hormone-binding globulin in a population survey in Sweden

**DOI:** 10.1530/EC-12-0057

**Published:** 2012-11-19

**Authors:** Bledar Daka, Thord Rosen, Per Anders Jansson, Lennart Råstam, Charlotte A Larsson, Ulf Lindblad

**Affiliations:** 1 Institute of Medicine University of Gothenburg PO Box 454SE-405 30, Gothenburg Sweden; 2 Department of Primary Health Care University of Gothenburg PO Box 454SE-405 30, Gothenburg Sweden; 3 Department of Endocrinology University of Gothenburg PO Box 454SE-405 30, Gothenburg Sweden; 4 Department of Internal Medicine University of Gothenburg PO Box 454SE-405 30, Gothenburg Sweden; 5 Department of Clinical Sciences Malmö Skåne University Hospital, Lund University Lund Sweden; 6 Social Medicine and Global Health LundSweden

**Keywords:** sex hormone-binding globulin, insulin, liver, diabetes

## Abstract

**Objectives:**

Obesity is associated with low levels of sex hormone-binding globulin (SHBG). While the reason is not fully understood, we aimed to study the association between serum insulin and levels of SHBG in a random population.

**Design and methods:**

Between 2001 and 2005, a random sample of 2816 participants aged 30–74 years were enrolled in a cross-sectional survey in the South-west of Sweden. Fasting blood samples were collected and an oral glucose tolerance test (OGTT) was conducted in all subjects without known diabetes. Diabetes mellitus was defined according to criteria from WHO, and clinical characteristics were used to discriminate between type 1 (T1D) and type 2 diabetes (T2D). Analyses of SHBG were successful in 2782 participants (98%), who thus constituted the current study population.

**Results:**

We found significant inverse association between levels of SHBG and fasting serum insulin in both genders (men: *β*=−0.090, *P*=0.001; women: *β*=−0.197, *P*<0.001), which was independent of differences in age and BMI. The associations remained when also differences in fasting plasma glucose were accounted for (men: *β*=−0.062, *P*=0.022; women: *β*=−0.176, *P*≤0.001). Subjects with T1D exhibited higher levels of SHBG than both T2D (men: *δ*=15.9 nmol/l, *P*<0.001; women: *δ*=71.1 nmol/l, *P*<0.001) and non-diabetic subjects (men: *δ*=15.1 nmol/l, *P*<0.001; women: *δ*=72.9 nmol/l, *P*<0.001) independent of age, BMI and fasting glucose levels.

**Conclusion:**

These findings are consistent with high levels of SHBG in T1D, and correspondingly low levels in T2D subjects, suggesting an inhibitory effect of insulin on the SHBG production in the liver.

## Introduction

Sex hormone-binding globulin (SHBG) is a circulating plasma globulin binding sex hormones, both oestradiol and testosterone and is produced primarily by the liver. In two recent studies, SHBG could predict type 2 diabetes (T2D) in both men and women (1, 2), and observational studies in men showed an association between SHBG and blood pressure (3). Moreover, early studies showed that low SHBG levels were associated with higher mortality in a cohort of postmenopausal women (4). Whether SHBG is a biomarker for obesity and the metabolic syndrome or if it is more directly implicated in the physiopathology of the metabolic syndrome is less clear. Interestingly, a specific membrane receptor for SHBG has been confirmed in different tissues (5). Thus, it seems that SHBG not only has a carrying function but also may exert direct cellular effects by modulating the target response of sex hormones.

Better knowledge of factors controlling the production of SHBG is important not least because of strong evidence for the relationship between SHBG and cardiovascular disease. Several factors have been suggested to influence the levels of SHBG (6, 7, 8). Obesity and hyperinsulinemia have for example been related to low levels of SHBG (9, 10). Moreover, *in vitro* studies have shown that insulin might inhibit production of SHBG in human hepatoma cells (11), and in accordance with these findings an inhibitory effect of insulin on SHBG secretion has been reported (12). In contrast with these findings, animal studies have shown that monosaccharides inhibit the gene that controls the production of SHBG (13) independent of insulin concentrations. However, it was unclear whether it is insulin or glucose, or both regulate the expression of the SHBG gene. Finally, a case–control study from Denmark has shown increased circulating concentrations of SHBG in men with type 1 diabetes (T1D) compared with healthy controls (14).

We aimed to study the association between endogenous insulin and SHBG-concentrations, and to estimate the differences in SHBG levels between subjects with T1D and T2D, and subjects without diabetes, respectively, in a population-based sample in South-western Sweden.

## Methods

### Subjects and methods

Between 2001 and 2005, a random population sample of 2816 men and women aged between 30 and 74 years, stratified by gender and by 5-year age groups, and who were residing in the municipalities of Vara and Skövde was enrolled in this study, with a participation rate of 76%.

The characteristics of the study population are described in detail in a previous publication (15). After exclusion of subjects without successful analyses for SHBG, 2782 subjects (men=1385; women=1397) remained to be analysed.

### Medical history, socio-economic and lifestyle factors

Standard questionnaires were used to obtain information on previous hospitalisations, current medication (including contraceptives and hormonal replacement therapy), smoking and alcohol habits as well as leisure time physical activity. Women on treatment with contraceptives and hormonal replacement therapy (progesterone alone, oestrogen alone or the combination) were excluded from analyses (remaining women=1002). Standard instruments were also used for the collection of data on demographic and socio-economic factors, and for symptoms of anxiety and depression (16).

### Clinical chemistry

Samples including plasma and serum were drawn after an overnight fasting and were immediately frozen at −82 °C. In participants without known diabetes mellitus, an oral glucose tolerance test (OGTT) was performed to characterize the participants with impaired fasting glucose, impaired glucose tolerance, and diabetes mellitus according to WHO criteria (17). Serum insulin was analysed using RIA. Insulin resistance was estimated using the homeostatic model assessment for insulin resistance (HOMA-IR) (18). Total testosterone was analysed according to UniCel DxI 800 Beckman Access Immunoassay System Main Instrument DxI-1 (19) in Malmö (Skåne University Hospital). Oestradiol and SHBG analyses were elaborated on at Unilabs at Skaraborg Hospital in Skövde, with results expressed in nanomole per liter (19). In 19 cases with SHBG levels reported to be higher than 180 nmol/l but not further specified, we assumed them to be 180 nmol/l. We calculated the free testosterone (FT) according to previous works (20, 21).

### Statistical analysis

Standard methods were used for descriptive statistics. General linear models were used to investigate the differences in SHBG levels between subjects with T1D, T2D, and those without diabetes respectively. The association between serum insulin levels and SHBG concentrations was studied by linear regression after excluding subjects on treatment with exogenous insulin and/or on hormonal replacement therapy. All statistical tests were two-sided and significance was accepted if *P*<0.05. SPSS Statistics Version 20 for Mac was used for all statistical calculations.

## Results

No gender differences were observed with regard to age and BMI, and the combined mean age was 47.8 years (±11.7 years) and mean BMI was 26.8 kg/m^2^ (±5.3 kg/m^2^). A strong inverse association was found in both men and women between SHBG and BMI, fasting plasma glucose, HOMA-IR and with serum triglycerides respectively (all *P*<0.001).

**Figure 1:**
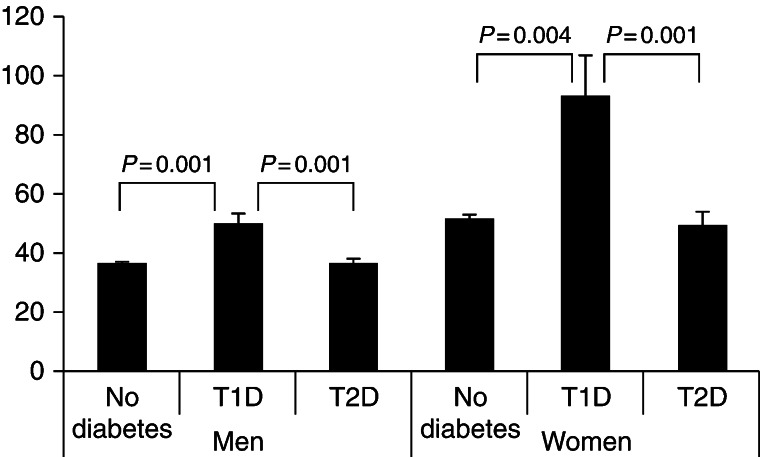
SHBG levels in healthy controls, type 1 and type 2 diabetes patients for both gender. General linear models were used and the results were adjusted for age, BMI, fasting glucose and free testosterone in general linear models.

T1D was diagnosed in 6 women and 12 men. When men and women were considered separately, SHBG levels were higher in subjects with T1D than in subjects without diabetes or those with T2D (Fig. 1). The difference was still statistically significant after adjustments for age, BMI, fasting glucose and triglycerides (*P*<0.001). Subjects with T2D had significantly lower levels of SHBG than subjects without diabetes when age adjusted analysis was computed separately for men and women. The differences remained after making further adjustments for BMI or triglycerides or both together. These differences were, however, no longer significant when fasting plasma glucose was included as a covariate.

The association between SHBG and fasting insulin in subjects without exogenous insulin is presented in Table 1. Fasting plasma insulin was significantly and inversely associated with SHBG concentrations independent of age, BMI and fasting glucose levels in both men and women. The association was stronger in women as it remained significant when triglycerides were also included into the model, while insignificance in men.

## Discussion

In this study of a Swedish adult population, subjects with T1D had significantly higher levels of SHBG than subjects without diabetes or with T2D. In addition, a strong inverse association between levels of SHBG and fasting endogenous serum insulin was found. Combined, these findings suggest an inhibitory effect of insulin in the production of SHBG by the liver.

Our results are consistent with previous smaller studies (2, 12, 14), including those with only men and with *in vitro* models (11, 13). In accordance with other studies, a strong association was found between fasting serum insulin, triglycerides and SHBG levels. Low SHBG levels seem to be a marker for the metabolic syndrome in both men and women. In T2D, the levels of SHBG were lower than in subjects without diabetes, but the association became insignificant after the adjustment for fasting glucose levels. This, combined with the strong association found between SHBG and glucose levels, suggests that glucose *per se* has a major role in the control of SHBG levels. In our population, the association between SHBG and insulin was significant and inverse in both men and women. While strong and robust in women, the association was somewhat weaker in men and became non-significant after adjusting for age, BMI, fasting plasma glucose and FT all together. The substantial reduction in the regression coefficient of serum insulin after adjusting for FT suggests a minor and non-significant inhibitory effect of insulin additional to FT on the control of SHBG production in men. However, the association remained significant and strong in women. In addition, the changes observed in the regression coefficient during adjustments for BMI and fasting plasma glucose suggest an active regulatory role by these variables in the control of SHBG levels.

The large population-based sample allowed for the investigation of this association in a representative population. Another strength was the accurate diagnosis of diabetes in accordance with WHO recommendation (17). Furthermore, a thorough medical history permitted for the exclusion of subjects on hormone medication (contraceptives or hormone replacement therapy), which could have influenced the association in the study. However, it should also be acknowledged that in this cross-sectional study the causality between SHBG and insulin could not be established.

In conclusion, these results suggest an inhibitory effect of insulin on the SHBG production. The effect of insulin on the SHBG production is of particular interest as low SHBG levels can, in part, predict the development of T2D and are associated with increased risk of cardiovascular disease and death. The possible direct effects of SHBG in these processes need to be investigated in more detail. In addition, these results will contribute to the understanding of regulation of SHBG levels in T1D and T2D.

## Author contribution statement

B Daka prepared the data, performed the statistical analyses, drafted the manuscript and took part in conceiving the study. T Rosen and P A Jansson participated in designing the study. C A Larsson worked on preparing data and offered expertise in statistical analysis. L Råstam conceived the study and acquired the data. U Lindblad conceived and coordinated the study, as well as acquired the data. He also participated in designing the study, performing statistical analyses and drafting the manuscript. All authors took part in designing the study, interpreting the data, revising the manuscript, and reading and approving the final manuscript.

## Figures and Tables

**Table 1:** The association between sex hormone-binding globulin (SHBG) and fasting insulin in men (*n*=1361) and women (*n*=1002) without diabetes.

	***β***	***P***
Men		
Adjusted for age	−0.251	<0.001
Adjusted for age and BMI	−0.090	0.001
Adjusted for age, BMI, F-PG	−0.062	0.022
Adjusted for age, BMI, F-PG, FT	−0.040	0.303
Women		
Adjusted for age	−0.368	<0.001
Adjusted for age and BMI	−0.197	<0.001
Adjusted for age, BMI, F-PG	−0.176	<0.001
Adjusted for age, BMI, F-PG, FT	−0.149	<0.001

Multivariate linear regression analysis of the association between SHBG and insulin. FPG, fasting plasma glucose; FT, free testosterone; *β*, standardised regressions coefficient. Subjects exposed to exogenous insulin or other sex-hormone therapy (preventive pills or hormone replacement therapy) were excluded from these analyses.
